# The Gingiva of Horses With Pituitary Pars Intermedia Dysfunction: A Macroscopic Anatomical Evaluation

**DOI:** 10.3389/fvets.2021.786971

**Published:** 2022-01-25

**Authors:** Anne Maria Nitzsche, Kerstin Fey, Kathrin Büttner, Manuela Gröf, Carsten Staszyk

**Affiliations:** ^1^Equine Clinic, Internal Medicine, Faculty of Veterinary Medicine, Justus-Liebig-University, Giessen, Germany; ^2^Unit for Biomathematics and Data Processing, Faculty of Veterinary Medicine, Justus-Liebig-University, Giessen, Germany; ^3^Faculty of Veterinary Medicine, Institute of Veterinary Pathology, Justus-Liebig-University, Giessen, Germany; ^4^Faculty of Veterinary Medicine, Institute of Veterinary-Anatomy, -Histology and -Embryology, Justus-Liebig-University, Giessen, Germany

**Keywords:** PPID, pituitary gland, gingival margin, periodontal disease, dental examination, equine

## Abstract

Pituitary pars intermedia dysfunction (PPID) is a common neurodegenerative disease mainly in horses older than 15 years. The domestic equine population is following the same demographic change as that seen in humans; it is aging and veterinarians are asked to attend to geriatric horses more frequently. Common problems seen regularly in older equines are dental disorders and especially periodontal disease. As a systemic and endocrine disease, associated with delayed wound healing and impaired immune function, PPID should be considered before major dental treatment in aged equines is started. Possible negative effects of PPID on epithelial tissues could also affect the periodontium. Therefore, the aim of the present study was to identify gross changes in the gingiva associated with PPID. Fourteen horses with clinical signs of PPID and adenoma in the pituitary pars intermedia and 13 controls showing neither clinical signs nor PPID-associated histological changes in the pituitary gland were included. PPID-affected horses (26.9 ± 0.73 years) were significantly older than controls (20.0 ± 1.24 years). In the PPID-affected group, significantly more often an irregular and bulky appearance of the gingival texture was observed, as well as an irregular shape of the gingival margin. Furthermore, the *sulcus gingivalis* of cheek teeth frequently was deeper than 1 mm. These findings indicate a possible association between age, soft tissue alterations, and PPID and suggest a potential predisposition of PPID-affected horses for periodontal diseases.

## Introduction

Pituitary pars intermedia dysfunction (PPID) is the most common endocrine disease of horses older than 15 years (1–4). It results from a slow degeneration of inhibiting dopaminergic neurons in the hypothalamus. The etiology is yet not fully understood, but oxidative stress and intra-neuronal α-synuclein deposition are the leading hypotheses (5, 6). The loss of the inhibitory function of dopamine on the melanotrophic cell-specific dopamine D2 receptor in the pars intermedia (PI) of the pituitary gland (7) leads to hyperplasia, micro- or macroadenoma, and an increased synthesis of the precursor protein proopiomelanocortin (POMC). POMC undergoes extensive tissue-specific posttranslational processing into smaller peptides by two serine proteases (prohormone convertase 1 and 2; PC1 and PC2) (8). PC1 is expressed in the pars distalis as well as in the PI of the pituitary gland, but PC2 is only expressed in the PI (4). The activity of PC1 and PC2 is also inhibited by dopamine (4). However, overall, the loss of dopaminergic neurons in PPID leads to increased activity of the melanotrophs, resulting in increased levels of PI-derived adrenocorticotropin (ACTH) and many other POMC-derived peptides, like α-melanocyte-stimulating hormone (α-MSH), corticotropin-like intermediate lobe peptide (CLIP), and β-endorphin in the systemic circulation. Due to individual increases in many of these POMC-derived peptides, the variance of clinical signs might be explained (6, 8–11). Typical clinical signs of PPID are long and curly hair known as hypertrichosis, abnormal hair shedding, wasting of the epaxial musculature leading to a potbelly appearance, abnormal fat distribution with periorbital fat deposition, hyperhidrosis, and—seldom—polydipsia and polyuria. However, the most dangerous and intolerable clinical sign of PPID is laminitis (2, 3, 11–19), from which about one-third of the PPID-affected horses will suffer (20).

“Equine Cushing Disease” is still a known term of the disease by owners, but it is important to notice that in contrast to the human or canine Cushing Syndrome, horses do not develop hypercortisolism (11, 21–23). The term PPID was introduced to emphasize that, in equines, melanotrophs are the cause of the clinically relevant overproduction of pituitary hormones (11). ACTH physiologically increases the production of cortisol in the cortex of adrenal glands and its concentration in peripheral blood. In PPID, even high peripheral concentrations of ACTH, measured by immunological tests, induce no functional relevant response of the adrenal gland and lead not to hyperplasia of the adrenal gland. Therefore, the so-called ACTH, produced by the cells of the PI, is considered to be not biologically active (24). Even though there is no hypercortisolism, there are clinical symptoms and changes in the endocrine system that would be well explained by effects of the glucocorticoid. One explanation could be that the normal circadian and annual rhythms of the cortisol concentration are lost (4, 21, 25, 26) and a relatively higher cortisol concentration occurs in PPID-affected horses (25). Another explanation would be a higher and faster cortisol turnover rate, leading to higher concentrations of metabolites in urine of PPID-affected animals (27). Additionally, horses with PPID produce higher levels of several immune-modulating or suppressing hormones like a-MSH, β-endorphin, and CLIP than non-affected adult, aged horses.

The effect of PPID on epithelial and connective tissues, especially its contribution to the development of laminitis or suspensory ligament degeneration, remains unclear (18, 24, 28). Corticotropin-like intermediate peptide (CLIP) is a cleavage product from ACTH and is increased in PPID. In genetically modified obese mice and pancreatic cells of rats, CLIP leads to an increase in insulin concentration (29, 30). Horses with insulin dysregulation are at high risk to develop laminitis. Experimentally induced hyperinsulinemia consistently induces acute laminitis in horses and ponies (20, 21, 26). Interestingly, 30–60% of PPID-affected horses show an insulin dysregulation (ID) (31, 32). Tadros and Frank (18) hypothesized that horses with PPID differ in types and amounts of POMC-derived peptides and those with additional ID may have higher ACTH-stimulated cortisol secretion. There are several studies trying to explain the influence of cortisol and insulin on basal epidermal cells of the hoof–lamellar interface, as yet however, none of them can fully explain the pathogenesis leading to laminitis (33, 34). Hyperadrenocorticism in other species leads to histologic alterations in the integument caused by protein depletion, inhibition of fibroblast growth, and reduction of collagen synthesis. Similar processes might result in weakening of the hoof structures in equines (18). More recently, the insulin-like growth factor-1 (IGF-1) was hypothesized to promote cell proliferation and structural weakening of the hoof lamellar suspensory apparatus (35).

The gingival epithelium is a major morphological and functional component of the dental supporting structures and there might be a link between PPID and equine periodontal disease. Gingival and periodontal structures in healthy horses were recently described from our group (36). Steinfort et al. (36) formed the basis to grade and evaluate equine periodontal disease, a common disorder especially of older equids (37–40). The periodontium consists of four different tissues, which form a structure for a healthy and stable tooth environment: alveolar bone, periodontal ligament, cementum, and gingiva (41). Each of these tissues has its own task in the hypsodont equine dentition (40). The early stages of periodontitis (gingivitis) are reversible and the regenerative capacity of the gingival epithelium is high, although deeper lesions, especially between teeth in interproximal/interdental spaces, are the onset of periodontal pockets and diastemata (38). The domestic equine population in Europe is following the same demographic change as that seen in humans—specifically, it is aging (42) and PPID and periodontal disease are both affecting mainly geriatric horses.

Clinical signs of PPID affecting the oral cavity or the periodontium are rarely reported, whereas sinusitis and non-healing ulcers in the labial part of the oral mucosa are described, as well as secondary dental diseases and apical infections (38, 43). PPID should be considered before major treatment of severe dental disorders in aged horses, because of possible impaired healing (5, 44). To the author's knowledge, there are no datasets or descriptions of gingival abnormalities in PPID-affected horses. We hypothesized that certain abnormalities of the gingival margin are altered in the presence of clinically appreciable PPID. Therefore, the aim of this study was to evaluate the gross anatomy of the gingiva of horses older than 15 years with and without PPID, in order to detect differences between these groups and possibly correlations between PPID and periodontal disease.

## Materials and Methods

A total of 85 horses and ponies of different breeds, aged ≥ 15 years, were included initially. They needed to be humanely euthanized for medical reasons unrelated to this study. The owner's consent was obtained by written information. They were asked about the medical and dental treatment history. The occurrence of hypertrichosis, a history of diagnosis, and/or treatment of PPID and clinical signs suspicious of PPID, e.g., prolonged shedding, generalized or local abnormal long hairs, laminitis, muscle atrophy leading to a “sway back” and “pot-belly” appearance, abnormal fat distribution, and/or hyperhidrosis, were recorded.

Within 72 h postmortem, the head was dissected from the body in the atlantooccipital joint. The pituitary gland was removed, and the weight, width, and length were measured. Afterwards, the pituitary glands were bisected in the median sagittal plane, and on the cut surface, the height was measured, before the left half was fixed in Klotz solution for photography and the right half was fixed in 10% neutral buffered formalin for histological examination. Tissue sections were prepared in a routine manner and stained with hematoxylin and eosin. The histopathological evaluation was performed by a blinded specialized veterinary pathologist (M. Gröf). Microscopical pituitary findings were scored according to the system of Miller et al. (17) depicting score 1: within normal limits; 2: focal or multifocal PI hypertrophy or hyperplasia; 3: diffuse PI adenomatous hyperplasia; 4: PI adenomatous hyperplasia with microadenomas (1 to 5 mm diameter); 5: adenoma (>5 mm diameter). Additionally, colloid-filled follicles (<1 mm in diameter) or cysts (≥1 mm in diameter) in the PI or pars distalis were noted.

According to history, and clinical and pathohistological findings, horses were grouped either as having PPID (*n* = 14) or controls (*n* = 13). Horses were included in the PPID group if they showed hypertrichosis or had a history of PPID with at least one clinical sign, and showed a histopathological score of at least 4 points. In the PPID group, 7 of 14 horses received treatment with pergolide. The control group consisted of horses showing no clinical signs or history of PPID and a microscopical score of not more than 2 score points (Table 1). The remaining 58 horses did not fulfill the inclusion criteria of either group and were excluded.

**Table 1:** Data of PPID and control horses examined in this study.

**No**.	**Group**	**Age** **[years]**	**Sex**	**Breed**	**Cause of euthanasia**	**Pergolide therapy**	**Clinical symptoms, associated with PPID**	**Pituitary Histology Score (17)**
1	PPID	20	Mare	Warmblood	Strangles	1 mg	Long coat, laminitis	5
2	PPID	24	Gelding	Warmblood	Intrathoracic neoplasia (lymphoma)	-	Partly long coat, focal hyperhidrosis, fetlock hyperextension	4
3	PPID	25	Gelding	Warmblood	Colic	n.a.	Hypertrichosis	5
4	PPID	25	Mare	Haflinger	Spine fracture	-	Longhairs on mandible, laminitis	5
5	PPID	26	Gelding	Icelandic horse	Colic	1 mg	Wintercoat in summer	4
6	PPID	27	Mare	Warmblood	Neoplasia bladder	1 mg	Partly longer coat, wasting of epaxial musculature	5
7	PPID	27	Gelding	Warmblood	Dysphagia	-	Hypertrichosis	5
8	PPID	28	Mare	Warmblood	Colic	2 mg	Laminitis, hyperhidrosis	4
9	PPID	29	Gelding	Warmblood	Colic	-	Hypertrichosis	5
10	PPID	29	Mare	Icelandic horse	Hoof abscesses	-	Hypertrichosis	4
11	PPID	29	Gelding	Pony	Colic	n.a.	Hypertrichosis	5
12	PPID	29	Gelding	Warmblood	Colic	-	Hypertrichosis	4
13	PPID	29	Mare	Pony	Colic	-	Hypertrichosis	4
14	PPID	30	Gelding	Pony	Laminitis	1.25 mg	Hypertrichosis, laminitis	5
15	Control	15	Gelding	Quarter Horse	Tendovaginitis	-	-	2
16	Control	15	Gelding	Draft	Myositis	-	-	2
17	Control	16	Gelding	Warmblood	Colic	-	-	2
18	Control	16	Gelding	Andalusian	Myositis	-	-	2
19	Control	18	Mare	Haflinger	Colic	-	-	2
20	Control	18	Mare	Draft	Myositis	-	-	2
21	Control	19	Mare	Icelandic horse	Colitis	-	-	1
22	Control	20	Mare	Icelandic horse	Strangles, cervical spine problems	-	-	2
23	Control	21	Gelding	Haflinger	Colic	-	-	2
24	Control	22	Mare	Friesian	Colic	-	-	1
25	Control	25	Mare	Warmblood	Colic	-	-	2
26	Control	26	Gelding	Icelandic horse	Colic	-	-	2
27	Control	29	Gelding	Pony	Colic	-	-	2

*-, no therapy; n.a., therapy but [mg/day] not assessable*.

The jaws were disarticulated at the temporomandibular joint and dissected with a band saw (type K440H, Kolbe Foodtec, Elchingen, Germany) on transversal planes to obtain the upper and lower incisor arcades as well as the upper and lower cheek teeth rows. Specimens were cleaned of loose food and debris with tap water and were fixed in 10% neutral buffered formalin. At each tooth position (TP) and interproximal position (IP), the adjacent gingiva was inspected on the vestibular and palatal/lingual side. The course of the gingival margin and the mucogingival junction (MGJ) was determined for the incisor arcade and cheek teeth row on the vestibular and palatal/lingual side. None of the cheek teeth specimens exhibited a first premolar. In total, there were 3,692 observed positions (OP), with 1,936 OP (1,056 TP/880 IP) in the PPID group and 1,756 OP (958 TP/798 IP) in controls. The following structures were inspected and scored with minor modifications as previously described by our group in Steinfort et al. (36).

### Gingival Sulcus

The presence of a gingival sulcus was assessed with a modified blunt measuring probe (DEN 30, Pferdefit-Dental, Hohenstein-Breithardt, Germany) featuring a millimeter scale. On each TP, the deepest point of the gingival sulcus was recorded as depth. According to Steinfort et al. (36), the following score was defined:

0: sulcus depth 0 mm to <1 mm (physiologic);
1: sulcus depth 1 mm to <3 mm (slightly abnormal);
2: sulcus depth ≥3 mm (pathologic).

### Periodontal Pocket and Diastemata

Periodontal pockets (PPs) were defined as macroscopically visible gaps between the tooth and the gingival margin and/or if food particles were present between the gingiva and tooth. The border between the physiological gingival sulcus and the pathologic PP was marked by an abrupt sulcular deepening. The PPs were further evaluated with the blunt measuring probe mentioned above. Location and maximal depth of the PPs were recorded. Any interproximal gap between two neighboring teeth, containing or not containing food, was defined as a diastema. Location and maximal depth of the diastema were recorded.

### Gingival Margin/Presence of a Papilla/Texture of Gingiva

The shape and contour of the gingival margin adjacent to a tooth were visually assessed and assigned to one of the following categories: regular or irregular. The texture (knots, cauliflower-like appearance) of the gingival margin, as well as its surface and smoothness, was described as gross changes visible or not. The presence or absence of interdental gingival papillae was recorded. The interdental papilla is the part of marginal gingiva that enters the interdental space.

### Mucogingival Junction

The MGJ was identified, according to the definition of Schroeder and Listgarten (45), as the border between the firmly bound gingiva and the movable alveolar mucosa. The MGJ was recorded on the vestibular side of the upper cheek teeth (UCT) and both sides of the lower cheek teeth (LCT). The MGJ is not present on incisors and on the palatal side of UCT.

### Peripheral Caries and Plaque (PCP)

Brownish discolorations of the teeth in association with pit-like depressions on the surface of the peripheral cementum were referred to as peripheral caries. Beige or yellowish hard deposits on the peripheral cementum were referred to as plaque. Presence and location of PCP were recorded.

### Statistical Analysis

The dataset of clinical and histological findings was analyzed descriptively. To detect differences between PPID and controls in age and pituitary gland parameters (weight, height, length, and width), the Wilcoxon–Mann–Whitney test was used as test for ordinal and metric variables in a two-group model, using the BMDP program package (Dixon, BMDP Statistical Software Manual, University of California Press, Berkeley, Los Angeles).

The datasets of the gingiva and teeth were analyzed with the statistical software SAS® 9.4 (SAS Institute Inc., 2008).

**Figure 1:**
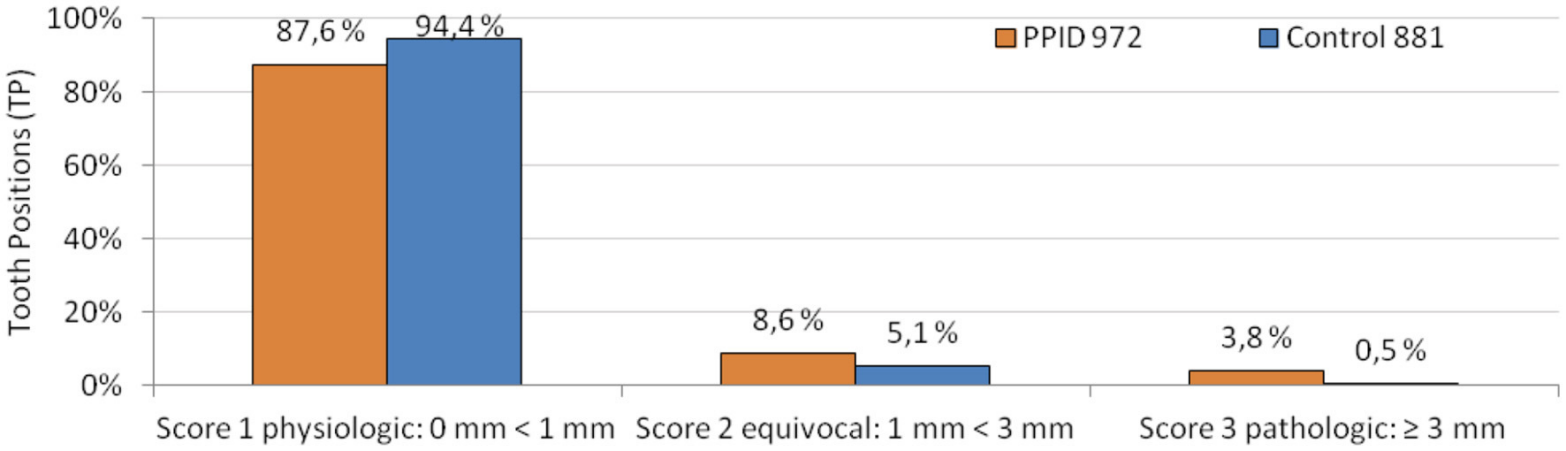
Gingival sulcus scores in PPID and control horses in relative numbers of all observed and measurable tooth positions. Absolute numbers of tooth positions are displayed behind the group legend.

As a first step, the dependence between tooth type (incisors or cheek teeth) and occurrence of changes from physiologic gingiva were calculated by the Chi-Square test. For all dependent variables (gingival sulcus score, PCP, pathologic alterations of gingival texture, PPs, diastemata, gingival margin, and MGJ), a significant association to the tooth type was found. Changes were seen in the majority of cases in the cheek teeth area. Therefore, the dataset was split and only the cheek teeth area was analyzed for all dependent variables mentioned above. To control for the confounding factor age, the covariate age as well as the interaction between age and group was included into the models. For gingival sulcus score and PCP, only positions on teeth were included in analyses, and for PPs and diastema, only interdental positions were included in analyses. For the pathologic gingival texture changes, the dataset was split and analyzed separately for tooth and interproximal positions. For the gingival margin and MGJ, a filtered dataset had to be used as both dependent variables were evaluated for the whole tooth arcade and not on single tooth and interproximal positions. The gingival sulcus was documented as multinominal data during data collection. Due to unevenly distributed data in the different gingival sulcus score categories (Figure 1), the scores were summarized to binominal categories (0 = no alteration, sulcus depth 0 < 1 mm; 1 = alteration, sulcus depth ≥ 1 mm) for analysis purposes.

#### Generalized Linear Models

All dependent variables were analyzed using the GLIMMIX procedure with a binomial distribution (link-function = logit). Fixed effects were added to the model in a stepwise manner. The Akaike's information criteria corrected (AICC) and the Bayesian information criteria (BIC) were used to compare the different models. The model with the smallest AICC and BIC values was chosen. All model results were calculated for a mean age of 23.6 years. The interaction between age and group was not significant for any of the dependent variables. Therefore, the interaction between age and group was excluded from the final model. In the following section, the specific models with the included fixed effects are explained.

In the dataset of the cheek teeth dental gingiva, the models for the dependent variables sulcus score, pathologic gingival texture changes, and PCP included the fixed effects group (PPID, controls), upper or lower cheek teeth (UCT, LCT) and tooth side (buccal or palatal/lingual), the interaction between group and UCT/LCT and between group and tooth side, as well as that between UCT/LCT and tooth side. The threefold interaction was tested, but was excluded from the model due to insignificance. The significance of differences in the least square means was adjusted by the Bonferroni correction.

In the dataset of the cheek teeth interdental gingiva (interproximal tooth spaces), the models for the dependent variables PPs and pathologic gingival texture changes included the fixed effects group (PPID, controls), upper or lower cheek teeth (UCT, LCT) and tooth side (buccal or palatal/lingual), and all two fold interactions between them. The three fold interaction was tested, but was excluded due to insignificance. The significance of differences in the least square means was adjusted by the Bonferroni correction.

The model for the dependent variable diastemata included the fixed effects group (PPID, controls) and upper or lower cheek teeth (UCT, LCT) and the interaction between them. The significance of differences in the least square means was adjusted by the Bonferroni correction. In the dataset of the whole cheek teeth arcade, the model for the dependent variables gingival margin and MGJ included the fixed effects group (PPID, controls) and upper or lower cheek teeth (UCT or LCT) and for the gingival margin also the tooth side (buccal or palatal/lingual) as well as all interaction between them. The significance of differences in the least square means was adjusted by the Bonferroni correction.

Results are presented as least squares means ± standard error (SE), if not mentioned otherwise. In all cases, a significance level of *p* ≤ 0.05 was used.

## Results

### Clinical and Pituitary Histological Findings

**Figure 2:**
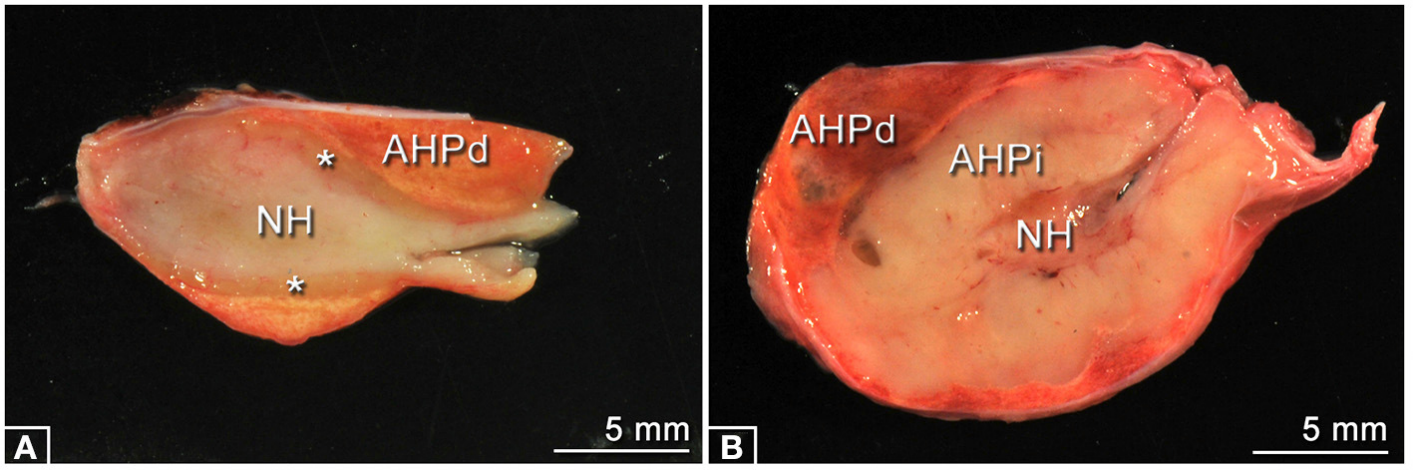
Typical examples of longitudinal midline sectioned pituitary glands of each group. **(A)** Specimen of the Control group, no pathological changes. The sizes of the neurohypophysis (NH), the adenohypophysis, pars distalis (AHPd), and the adenohypophysis, pars intermedia (asterisks) are in normal appearance ranges. **(B)** Specimen of the PPID group showing a massively enlarged adenohypophysis, pars intermedia (AHPi).

The PPID group consisted of 14 horses that met the inclusion criteria and 13 horses were classified as controls. Ages in the PPID group ranged from 20 to 30 years (mean ± standard deviation 26.9 ± 0.73 years) and those in controls ranged from 15 to 29 years (20.0 ± 1.24 years); the PPID horses were significantly (*p* = 0.0006) older than controls. Therefore, results were computationally adjusted before statistical analysis. The sex distribution was nearly similar in both groups: 8 geldings were included in the PPID group, 7 geldings in the control group, and 6 mares in each group. In the PPID group, horses were mainly warmbloods 57.1% (8/14), followed by three ponies, two Icelandic horses, and one Haflinger. The controls were a mix of different breeds (three Icelandic horses, two draft horses, two warmbloods, two Haflinger, and one Pony, Quarter Horse, Friesian, and Spanish Horse each). Individual signalment (age, breed and sex), results of clinical examination, reported therapy with pergolide, reason for euthanasia, and pituitary gland scores are displayed in Table 1. Single or multiple follicles in pituitary glands were seen in 92.9% (13/14) of PPID-affected horses, while 69.2% (9/13) of the controls showed follicles. Single or multiple cysts were recorded in the PPID group in 85.7% (12/14) in contrast to 46.2% (6/13) of the control group. The pituitary glands showed significant differences between the PPID and control group (Figure 2, Table 2) in pituitary weight (*p* = 0.0001), height (*p* = 0.001), and length (*p* = 0.018).

**Table 2:** Pituitary gland findings of PPID and control horses (mean ± standard deviations).

Pituitary gland histology score^*^	**Group**	***n***	**Age [years]**	**Pituitary gland weight [g]**	**Pituitary gland height [cm]**	**Pituitary gland length [cm]**	**Pituitary gland width [cm]**
1	Control	2	19, 22	1.54, n.a.	0.75 ± 0.07	1.70 ± 0.28	1.50 ± 0.28
2	Control	11	19.9 ± 4.83	2.24 ± 0.80	0.82 ± 0.20	2.10 ± 0.37	2.16 ± 0.36
4	PPID	6	27.5 ± 2.07	4.23 ± 1.41	1.28 ± 0.58	2.38 ± 0.62	2.18 ± 0.29
5	PPID	8	26.5 ± 3.21	7.72 ± 4.35	1.89 ± 0.71	2.70 ± 0.71	2.60 ± 0.50

* *Scored according to Miller et al. (17). Score 3 is lacking because horses with 3 points were not included in the study. n.a., not applicable (weight of pituitary gland of horse no. 21 is missing)*.

### Overview of Results Concerning Gingival Findings and Peripheral Caries/Plaque

Table 3 summarizes the results of all investigated parameters after adjusting the raw data to a mean age of 23.6 years in both groups.

**Table 3:** Differences between PPID and controls in parameters of interest.

**Abnormal findings in cheek teeth**	**PPID group**	**Control group**	***p*-value**
Irregular texture of dental gingiva	43.9% (296/656)	18.3% (121/606)	**<0.0001**
Gingival sulcus > 1 mm	17.6% (113/604)	7.6% (48/557)	**0.004**
Irregular texture of interdental gingiva	45.2% (255/544)	35.3% (171/504)	**0.022**
Irregular gingival margin	65.6% (81/112)	43.2% (38/102)	**0.040**
Diastemata	16.2% (160/544)	19.8% (70/504)	0.241
Irregular mucogingival junction	39.9% (38/83)	34.9% (19/76)	0.698
Peripheral caries and plaque	15.3% (105/656)	33.0% (223/606)	**<0.0001**
Periodontal pockets	8.9% (76/544)	14.8% (57/504)	**0.023**

*Percentages and p-values are given after computational adjustment of data to the mean age of 23.6 years in both groups. Absolute numbers of findings/observed positions are given in brackets*.

*Significant p-values are presented in bold letters*.

### Gingival Margin/Presence of a Papilla/Texture of Gingiva

**Figure 3:**
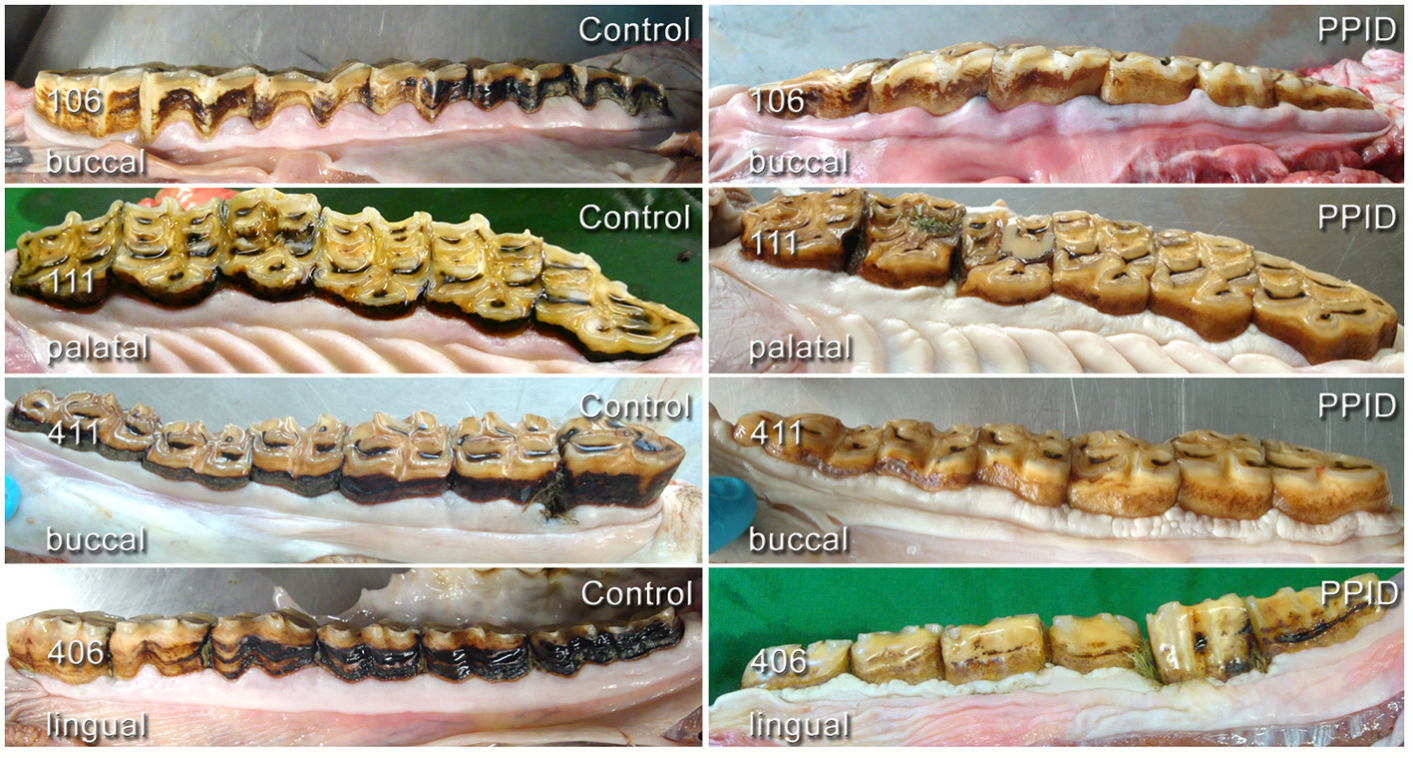
Comparison of gingival shape and contour in cheek teeth rows of healthy (left column) and PPID-affected horses (right column). Note the changes in tissue texture and irregularities in the course of the gingival margin.

The normal “garland-shaped” gingiva of the incisors, as described by Steinfort et al. (36), was found in both groups in every position (buccal/labial and palatal/lingual) and an interdental papilla was always present. The cheek teeth rows of PPID horses had in 65.6% an irregular course of the gingival margin, compared to control horses, which showed this feature in 43.2%. The presence of an irregular gingival margin in the cheek teeth area was significantly (*p* = 0.008) associated with age and showed a significant interaction between the group and the toothside (*p* = 0.05). The specific gingival margin of the buccal side of the UCT with the regular “double-waved” appearance and the almost straight-lined gingival margin of the buccal side in LCT was not present in 76.5% of PPID and in 72.1% of controls. The gingival margin of the palatal tooth side in UCT and lingual tooth side in LCT showed a significant (*p* = 0.03) difference between the groups and was irregular in 52.9% of the PPID group and in 18.4% of the controls (Figure 3). Only within the control group did the buccal tooth side show significantly (*p* < 0.0001) more often an irregular gingival margin than the palatinal/lingual tooth side.

**Figure 4:**
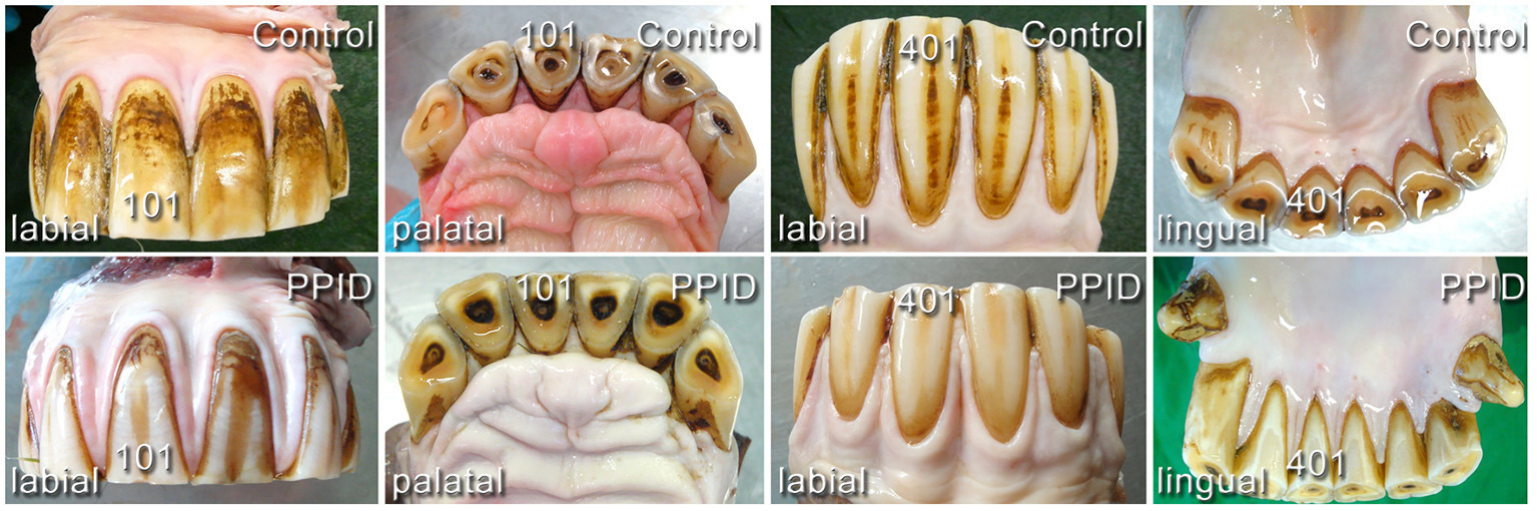
Comparison of gingival shape and contour in incisor arcades of healthy (upper line) and PPID-affected horses (lower line). No visible macroscopical differences.

On incisors, the presence of uneven, rough, knot-like and bulky irregular gingival texture (Figure 4) was recorded in 19.0% (140/736) of the PPID and 13.5% (87/646) of the control specimens. An irregular gingival texture of cheek teeth dental positions was exhibited in 43.9% of PPID-affected horses, compared to control horses featuring an irregular gingival texture of cheek teeth dental positions in 18.3% (*p* < 0.0001; OR: 3.49). The interaction between group and tooth side (*p* = 0.007) showed a significant influence on the presence of irregular gingival texture. Within each group, the buccal cheek teeth sides showed more irregular gingival texture than the palatal/lingual sides (PPID *p* = 0.0003; control *p* < 0.0001). The PPID group showed more irregular gingival texture than the control group for both cheek teeth sides (buccal toothside *p* = 0.0003; palatinal/lingual toothside *p* < 0.0001). Other interactions between the fixed effects were not significant.

An irregular gingival texture of cheek teeth interdental positions was exhibited in 45.2% of PPID-affected horses, compared to control horses featuring an irregular gingival texture of cheek teeth interdental positions in 35.3% (*p* = 0.02; OR: 1.51). The presence of an irregular gingival texture on cheek teeth position (on the interdental papillae) showed a significant interaction between the UCT/LCT and the tooth side (*p* = 0.02). The buccal LCT interdental positions showed significantly more irregular gingival texture than the lingual LCT interdental positions (*p* < 0.0001). With 53.4%, the LCT on the vestibular side showed a significantly (*p* = 0.015) higher presence of irregular gingival texture on interdental positions than the UCT vestibular sides with 40.0%. This difference between UCT and LCT could not be seen on the palatal/lingual tooth side. Other fixed effects and two fold interactions were not significant.

### Gingival Sulcus

Gingival sulci ≥ 1 mm were present in 17.6% of investigated tooth positions in PPID-affected horses and in 7.7% of investigated tooth positions in control horses (*p* = 0.004; Table 3). The buccal tooth side of the cheek teeth area showed a higher presence of gingival sulci ≥ 1 mm than the palatinal or lingual side (*p* = 0.002; OR: 1.86). The interaction between the groups and the upper cheek teeth (UCT)/lower cheek teeth (LCT) for the presence of gingival sulci ≥ 1 mm was significant (*p* = 0.003). In the PPID group, no significant difference between the UCT and the LCT was found, while the control group showed significantly more gingival sulci ≥ 1 mm in the UCT than in the LCT (*p* = 0.02). Within the UCT, 16.4% of PPID and 12.0% of the controls showed gingival sulci ≥ 1 mm, but this difference between the groups was not significant. In the LCT, the PPID group showed with 18.9% a significantly (*p* = 0.0002) higher presence of gingival sulci ≥ 1 mm than the controls with 4.8%.

### Diastemata

**Figure 5:**
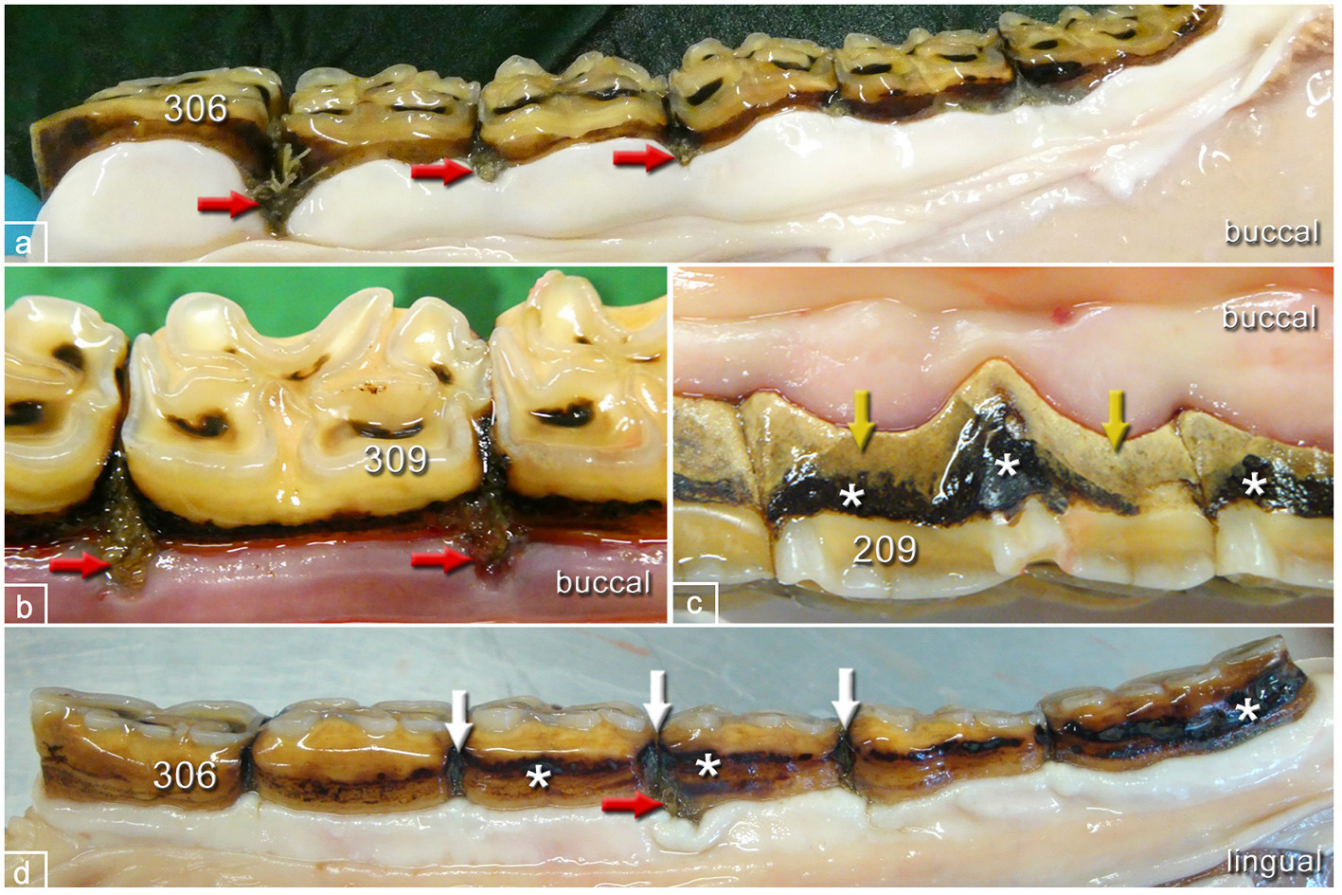
Typical pathological changes in horses of the control group. **(a)** Left lower jaw, buccal aspect, 22-year-old mare showing multiple widened interproximal spaces with pocket formation and food entrapment (red arrows). **(b)** Left lower jaw, buccal aspect, 19-year-old mare showing widened interproximal spaces with pocket formation and food entrapment (red arrows). **(c)** Left upper jaw, buccal aspect, 16-year-old gelding showing peripheral carious lesions (asterisks) and tartar (yellow arrows) but no pocket formation. **(d)** Left lower jaw, lingual aspect, 20-year-old mare showing peripheral carious lesions (asterisks), widened interproximal spaces (white arrows), and pocket formation (red arrow).

**Figure 6:**
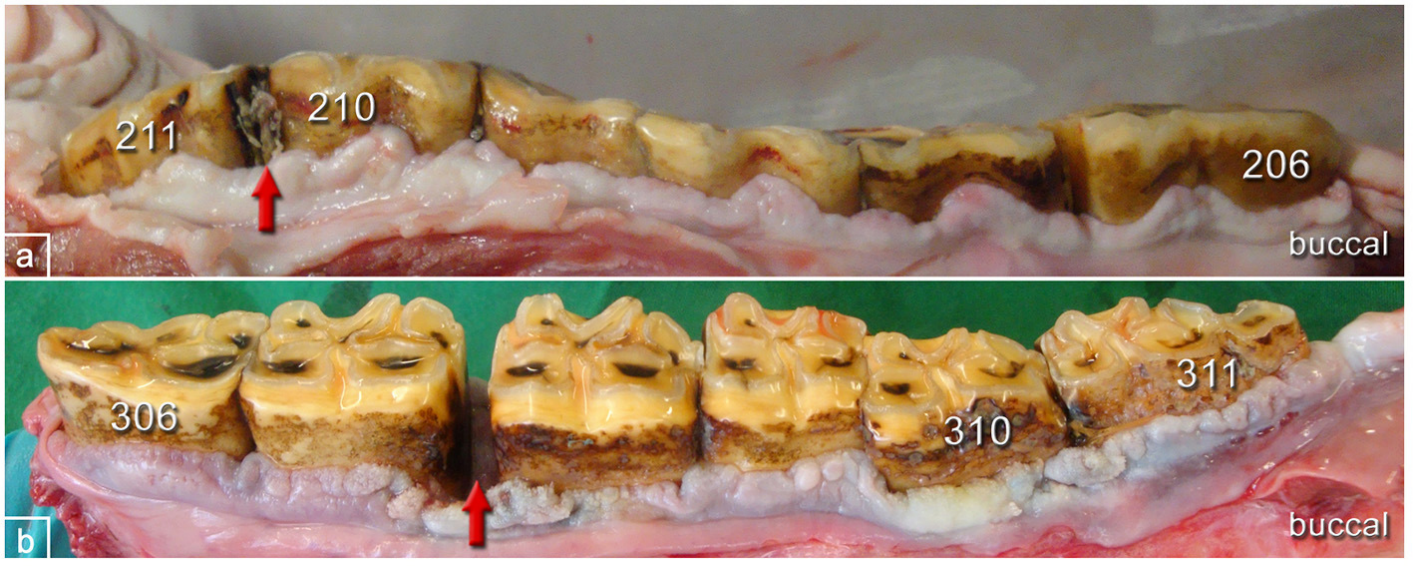
Typical pathological changes in horses of the PPID group. **(a)** Left upper jaw, buccal aspect, 27-year-old gelding showing gingival texture alterations and irregular contour of the gingival margin. Red arrow: widened interproximal space and food entrapment. **(b)** Left lower jaw, buccal aspect, 28-year-old mare showing gingival texture alterations and irregular contour of the gingival margin. Tooth 310 is shifted buccally. Red arrow: widened interproximal spaces with pocket formation.

When controlling for age, PPID horses exhibited in 16.2% of cheek teeth interdental positions diastemata, compared to control horses that showed diastemata in 19.8% of cheek teeth interdental positions (*p* = 0.2). At least one diastema was diagnosed in each horse (Figures 5, 6). The presence of diastemata was significant (*p* < 0.0001) associated with age. In both groups, the LCT showed more frequently (*p* = 0.0006; OR: 1.8) diastemata (22.7%) than the UCT (14.0%). Triadan positions 406/407 and 306/307 were the most frequent locations for diastemata in both groups.

### Mucogingival Junction

The cheek teeth rows of the PPID group exhibited in 39.9% an irregular MGJ, compared to controls, which exhibited an irregular course of the MGJ in 34.9% (*p* = 0.7). Irregular MGJ correlated with age (*p* = 0.02) and the UCT of both groups showed with 63.5% a higher presence (*p* < 0.0001) of irregular MGJ than the LCT with 17.0%. The interactions between groups and UCT/LCT were not significant (*p* = 0.07).

### Peripheral Caries and Plaque

Peripheral caries and plaque were seen in 15.3% of dental tooth positions in the PPID group, compared to control horses, which exhibited peripheral caries and plaque in 33.0% of dental tooth positions (*p* < 0.0001). Age showed a significant influence on the presence of PCP (*p* = 0.02). Younger animals showed a higher OR to develop PCP of 1.04 than older horses with an OR of 0.96. All two fold interactions between the groups and UCT/LCT (*p* = 0.04), groups and cheek teeth side (*p* = 0.02), and UCT/LCT and cheek teeth side (*p* = 0.05) were significant concerning the presence of PCP on cheek teeth. The PPID group showed a higher (*p* = 0.01) presence of PCP in UCT (20.7%) than in the LCT (11.7%). In controls, there was no significant difference between UCT and LCT. Within the UCT (*p* = 0.01) and within the LCT (*p* < 0.0001) and also within the buccal (*p* = 0.02) or palatal/lingual cheek teeth side (*p* < 0.0001), the control group showed more PCP than the PPID group. For both groups, the buccal cheek teeth side showed a higher presence of PCP than the palatal/lingual cheek teeth side (PPID *p* < 0.0001, controls *p* = 0.005).

### Periodontal Pockets

**Figure 7:**
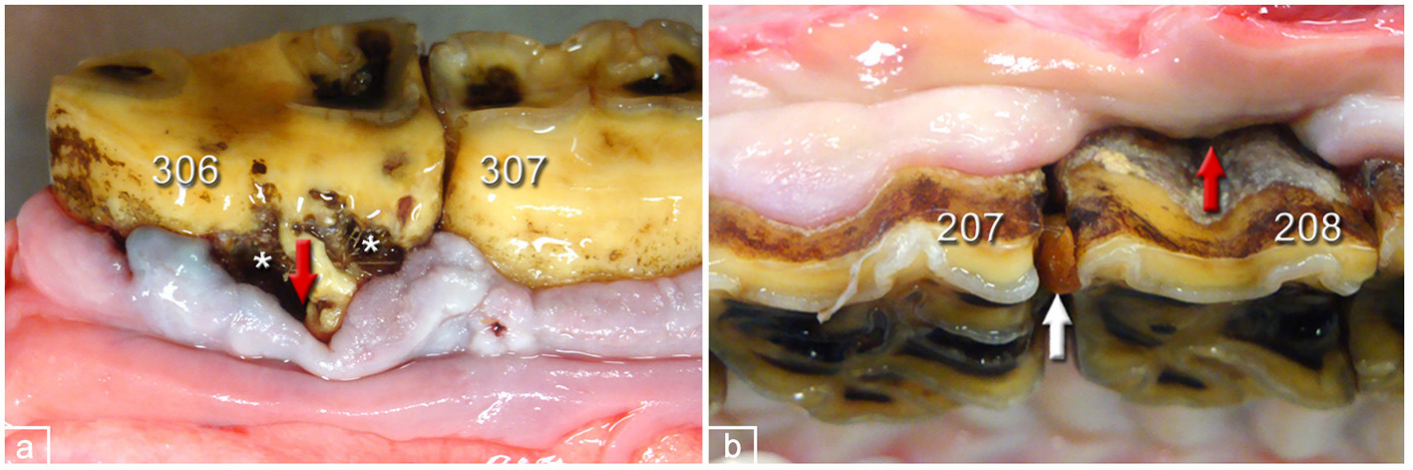
Periodontal pockets in dental gingiva of PPID-affected horses. **(a)** 306, buccal aspect. Peripheral carious lesions (asterisks), pocket formation (red arrow), and gingival hyperplasia. **(b)** 208 buccal aspect. Gingival regression, pocket formation (red arrow), and interproximal food entrapment (white arrow).

Periodontal pockets were found in cheek teeth interdental positions of PPID-affected horses in 8.9%, compared to control horses, which exhibited PPs in cheek teeth interdental positions in 14.8% (*p* = 0.02). The presence of periodontal pockets was significantly (*p* < 0.0001) associated with age. In each group, older animals had a higher chance to develop PPs (OR: 1.15). After computationally controlling for age, controls showed a higher OR of 1.76 to develop PPs than PPID-affected horses with an OR of 0.57. The PPs were visible without manipulation and located interproximally, except for 2 PPs that were found adjacent to a tooth (Figure 7). Altogether, 93.5% (129/138) showed food impaction and 72.5% (100/138) were associated with diastemata. PPs were found in 9.7% of the UCT and in 13.6% of the LCT interproximal regions (*p* = 0.05). The tooth side and all two fold interactions between the fixed effects were not significant.

## Discussion

To the author's knowledge, this study reports findings of the equine gingiva in aged horses with and without PPID for the first time. Age had no influence on gingival texture alterations or on presence of gingival sulci ≥ 1 mm depth, and both alterations were found more frequently in the PPID group than in controls. On the other hand, peripheral caries and plaque as well as periodontal pockets were seen more often in controls than in horses affected by PPID.

### Clinical and Histological Findings in the Pituitary Gland

Methodologically, two critical points have to be addressed: there was a statistically significant difference between the mean ages (PPID: 26.9; controls: 20.0 years) of the groups. However, to the author's knowledge, this is the first study to address gingival tissue and tooth problems in PPID and in a geriatric control group. The interaction between age and group was not significant for any of the tested dependent variables. Accordingly, the changes in both groups behave similarly with regard to age and one can concentrate on the main effects. However, as this is a clear limitation of this study, we would recommend selecting evenly aged groups for future studies.

The PPID group consisted of mainly warmbloods (57.1%) while the control group included a mix of different breeds and only two warmbloods. There are very few studies on equine gingiva and none of them compared the gingiva of different breeds. For the evaluation of the physiological gingiva, mainly warmbloods (75%) were used (36). However, all equine breeds show the hypsodont remodeling dentition and tooth anatomy principally remains the same. Therefore, we do not expect major differences between different breeds, but we recommend selecting even better for age and breed matched groups for further research.

The histological findings of adenomatous hyperplasia, micro- or macroadenomas in the PI are considered to be the gold standard for the diagnosis of PPID (17). However, age-related changes in the pituitary gland and in the PI are common findings in old horses (14, 19, 46, 47). In 17% of horses without clinical symptoms for PPID, van der Kolk et al. (47) found micro- or macroadenoma in the PI and their occurrence was significantly associated with age. This displays the difficulties in diagnosing PPID, being a slowly developing and progressing neurodegenerative disease, even with the help of pituitary gland histology. Therefore, we decided to include in the PPID group only horses with clinical symptoms for PPID and micro- or macroadenoma in the PI with a score of 4 or 5 points. In contrast, controls needed to have shown no signs suspicious for PPID and a PI score of 1 or 2 points. The latter is the main reason why we could not select higher numbers of control horses in our study. However, due to these strict inclusion criteria, we generated clearly discriminated groups.

### Texture of Gingiva

To the author's knowledge, no other group focused on the gingival texture in horses before. The gingival margin is physiologically flat and shows a smooth and even surface. However, in PPID-affected horses, visible changes in the gingival texture leading to an uneven surface were frequently (43.9%) recorded. These changes were described as hyperplastic gingiva. In a histopathological study of Cox et al. (48), gingival hyperplasia was a very common finding, affecting each investigated horse to some extent. The authors explained their findings by the coarse feed material. Such an irregular gingival texture was found in the control group in our study less frequently (18.3%) and exclusively with concomitant periodontal pathologies such as PPs or diastemata. It is assumed that these caused the gingival texture changes by inflammatory stimuli. No influence of age on gingival texture changes was detected. In the PPID group, not only did gingival texture changes occur in higher numbers on both tooth sides (buccal 51.9%, palatinal/lingual 36.2%; *p* < 0.001) compared to the control group (buccal 31.2%, palatinal/lingual 10.0%), but they were also seen without co-existing other periodontal pathologies. This finding might reflect a systemic impact of PPID on the gingival tissue. This assumption is supported by the description of similar gingival texture changes in men suffering from diabetes mellitus (49).

### Gingival Sulcus

More than 91% of the controls showed sulcus depths of <1 mm. This is a physiological finding and in accordance with the investigations of Steinfort et al. (36). To the author's knowledge, there is no description of increased sulcus depth associated with Cushing's syndrome in human or canine dental medicine literature. In the PPID group, only 81% showed sulcus depths of <1 mm. Deepened sulci were evenly distributed over the arcades in the PPID group, whereas the few sulci ≥ 1 mm in controls were seen predominantly in upper cheek teeth (*p* = 0.02) on both sides, and almost none on the lingual side of lower cheek teeth (LCT). These results confirm the findings of Steinfort et al. (36). They found sulci ≥ 1 mm more frequently on the palatal side of UCT and on the buccal side of LCT. The uneven localization of deepened gingival sulci in the control group might reflect high biomechanical stimuli on the palatal side of UCT and the buccal side of LCT. In contrast, PPID-affected horses show many more deepened gingival sulci in all localizations. We assume that systemic influences cause sulcus alterations additionally to the biomechanical effects. Macroscopically detectable attachment loss is described as onset of periodontal disease in human and canine dental medicine (50). This allows food and debris to enter the sulcus and to remain for longer time periods, leading to structural damage of the gingival barrier. This pathogenesis could potentially predispose to the onset of periodontal disease in PPID.

### Gingival Margin/ Presence of Papilla/MGJ

The examination of the complete dental arcade might be challenging in living horses, especially for the buccal aspect of the caudal cheek teeth. Knowledge of the physiological shape and contour of the gingival margin is the basis to detect alterations and draw conclusions on gingival health. In controls, the shape and contour of the gingival margin varied physiologically within the dental arcades as it was described by Steinfort et al. (36). Fiorellini et al. (51) concluded that the margin follows the shape of the embrasures of the tooth surface, the shape of the teeth, and their alignment in the arch. Nevertheless, the contour of the UCT buccal part was irregular in 50.4% of the control horses and in more than 68.5% in horses of the PPID group. The reason for the irregular shape of the gingival margin was that more than 75% were periodontal pathologies (e.g., diastemata, periodontal pockets, and gingival recession; PPID: 72/134; control: 30/134). No visible reason was found in 17/134 PPID-affected horses and 15/134 controls. In the control group, irregularities (and missing interdental papilla) were found almost exclusively on buccal sides of cheek teeth, which was consistent with the findings of Steinfort et al. (36). We report here for the first time that specimens of PPID-affected horses showed markedly more irregular shapes and contours of the gingival margin in all four arcades and sides of cheek teeth. Since age significantly increased the occurrence of an irregular gingival margin, it remains unclear to what extent PPID causes the described alterations of the gingival margin within our study population. However, after statistically adjusting for age, the highly significant increased occurrence of irregularities in PPID clearly remained. The MGJ is used in human and canine dentistry as an important landmark for measuring gingival recession and for planning surgical procedures. As Steinfort et al. (36) already indicated, it is not possible to transfer this to equine dentistry. The MGJ is not visible in all locations (e.g., the palatal side of UCT and the lingual aspect of the incisors) and a cementoenamel junction is absent. Therefore, we did not evaluate the presence of the MGJ on incisors in this study and evaluated only clearly visible cheek teeth positions. Irregular MGJ was found significantly (*p* < 0.0001) more often in UCT than in LCT. There was a significant influence of age (*p* = 0.02) but not of group. The irregular appearance of the MGJ was regularly associated with periodontal pathologies and major changes in the gingival margin.

### Diastemata

Numerous diastemata (77.4%) found in this study were located between cheek teeth, which coincides with current literature (36, 52). The results of our study are consistent with several publications, that diastemata are mainly localized in the lower cheek teeth region and the prevalence increases with age (36, 52–56). Senile diastemata can occur as a function of tooth shape (43). We could not show that PPID has a direct influence on diastema occurrence.

### Peripheral Caries and Plaque

Peripheral caries and plaque were found with a prevalence of 26% (328/1262 TP). The prevalence of peripheral caries is quite different: studies from various countries reach from 6.1% in Sweden (57) up to 58.8% in Australia (58). Although widespread in the equine population, peripheral caries, dental calculus and plaque are not considered a significant problem in equine cheek teeth because of the eruption and replacement by unaffected tooth substance (38, 55, 59). The location of peripheral caries is described to be mostly on the three caudal maxillary or mandibular cheek teeth (55, 60). This can be explained on one hand by the anatomical location of the ductus parotideus, entering the mouth medially and next to the fourth premolar in UCT. Since it drains rostrally, the saliva coats the premolars and provides a protective environment for these teeth. On the other hand, the masticatory process moves food caudally in the mouth, breaking it down in smaller particles, therefore making the food more accessible for cariogenic bacteria. Bacterial metabolism of carbohydrates produces organic acids, resulting in a lowered biofilm pH and finally in demineralization of cementum (59, 61). In both groups, PCP were seen more often on the vestibular tooth side (PPID *p* < 0.0001; controls *p* = 0.005). In the control group, PCP were evenly distributed between the UCT (118 TP, 34.4%) and LCT (105 TP, 31.6%). This is in accordance with the findings of Ramzan et al. (55), who found peripheral caries evenly distributed between the maxillary (46.5%) and mandibular (53.5%) cheek teeth rows. Jackson et al. (61) found moderate or severe peripheral caries within the mandibular rather than the maxillary cheek teeth row. In our PPID group, PCP were found more often (*p* = 0.01) in UCT (66 TP, 20.7%) than in LCT (39 TP, 11.2%). This might be caused by the overall low prevalence of PCP in our cheek teeth material. We did not differentiate between peripheral caries, dental calculus and plaque, leading to a potential higher occurrence of PCP. Interestingly, the control group showed a higher number of PCP (33.0%) than the PPID group with 15.3%, which differs from Simon and Herold (62), who propose that PPID is leading to immunosuppression and results in plaque formation and chronic gingivitis. Lee et al. (63) found a significant, negative association between age and the prevalence of peripheral caries. In their study, every horse between 11 and 20 years was affected by peripheral caries, while horses over 20 years showed a prevalence of 89.7%. In our aged study population, the ORs for horses of <23.6 years (1.04) differed not considerably from older probands (OR: 0.96).

### Periodontal Pockets

In human dental medicine, a periodontal pocket is defined as an abrupt “pathologically deepened” gingival sulcus (50). A “clear distinction between a PP and the physiological gingival sulcus is mandatory to distinguish between normal anatomical variation and a pathological condition” (36). We used the same definition of PPs as previous studies: a macroscopically visible gap between the gingiva and the tooth with an abruptly deepened gingival sulcus. The gingiva is not any longer in line with the rest of the gingival margin, forms a groove or depression (“pocket”), and detaches from the tooth. PPs in our controls were located exclusively interproximally, which was seen by Cox et al. (48) and Steinfort et al. (36) too. Only in the PPID group were two PPs located in the non-interproximal buccal dental gingiva. This might reflect a weakened gingival margin and a predisposition for periodontal disease. In contrast to the horse, PPs in humans emerge everywhere throughout the gingival margin (50). The etiopathogenesis of PPs in the hypsodont dentition of the horse differs from brachydont species. In men and dogs, the accumulation of plaque and bacteria frequently results in gingivitis, which initially leads to a deepened gingival sulcus and severe periodontal disease (50). In hypsodont species, the main reason for the occurrence of PPs is food entrapment in widened interproximal positions, causing the formation of diastemata and deep interdental pockets. The role of plaque and bacteria in the etiopathogenesis of PPs are assumed to play a minor role in equines (55, 61). The seldom occurrence of PPs in non-interdental gingiva locations in horses might be explained by the continuous tooth eruption, which triggers a continued remodeling of the gingival tissues. Our findings of PPs predominantly in older animals of both groups and with higher numbers in LCT than UCT are in accord with current literature (48, 64). We could not show an influence of PPID on the presence of PPs. Interestingly controls showed even a higher odds ratio to develop PPs. This might be an effect of the also higher prevalence of plaque and caries in the control group. Equine peripheral caries causes cemental destruction, opens a formerly closed interproximal/interdental position, and therefore predisposes for the development of PPs.

## Conclusions

For the first time, macroscopic gingival alterations in horses with PPID compared to controls are described. The increased occurrence of gingival texture changes, irregular gingival margins, and deepened gingival sulci suggest a potential predisposition of PPID-affected horses for periodontal disease. Endocrinological pathways, which could explain the systemically weakened gingival tissue structure in PPID, need to be explored.

## Data Availability Statement

The raw data supporting the conclusions of this article will be made available by the authors, without undue reservation.

## Ethics Statement

Ethical review and approval was not required for the animal study because according to German legislation, the post mortem collection of specimens does not need permission of the animal welfare authority. Written informed consent was obtained from the owners for the participation of their animals in this study.

## Author Contributions

AN wrote the manuscript and performed the macroscopic evaluations of the gingiva. MG performed the histopathological scoring of the pituitary glands. CS and KF contributed to conception and design of the study. KB performed the statistical analysis. All authors read the manuscript, contributed to manuscript revision, and approved the submitted version.

## Funding

This study received funding from Boehringer Ingelheim Vetmedica. The funder was not involved in the study design, collection, analysis, interpretation of data, the writing of this article or the decision to submit it for publication.

## Conflict of Interest

KF has a consultancy agreement with Boehringer Ingelheim Vetmedica. The remaining authors declare that this study was conducted in the absence of any commercial or financial relationships that could be construed as a potential conflict of interest.

## Publisher's Note

All claims expressed in this article are solely those of the authors and do not necessarily represent those of their affiliated organizations, or those of the publisher, the editors and the reviewers. Any product that may be evaluated in this article, or claim that may be made by its manufacturer, is not guaranteed or endorsed by the publisher.
