# Supplementary material for: The Gingiva of Horses With Pituitary Pars Intermedia Dysfunction: A Macroscopic Anatomical Evaluation
Source: Front Vet Sci. 2022 Jan 25;8:786971. doi: 10.3389/fvets.2021.786971 (PMC8821874; doi:10.3389/fvets.2021.786971)
Supplement: Supplementary file 1 [file Data_Sheet_1.PDF]

Table 4: Coefficients, SE, and 95% CI of variables included in the model regarding gingival texture changes at dental gingival positions

			95% Confidence Limits		
	Coefficient	SE	Lower Bound	Upper Bound	*global* *P*-value
AGE	0.01362	0.01904	-0.02373	0.05097	0.4745
GROUP					<.0001
PPID	0.9283	0.2547	0.4286	1.428	
Control	0				
UPPER CT/ LOWER CT					0.3107
UCT	0.381	0.2255	-0.06142	0.8234	
LCT	0				
TOOTH SIDE					<.0001
palatinal/lingual tooth side	-1.2217	0.27	-1.7514	-0.6919	
buccal tooth side	0				
GROUP*UCT/LCT					0.6569
PPID*UCT	-0.1198	0.2697	-0.649	0.4093	
PPID*LCT	0				
Control*UCT	0				
Control*LCT	0				
GROUP* TOOTH SIDE					0.0069
PPID*inner tooth side	0.7615	0.2817	0.209	1.3141	
PPID*buccal tooth side	0				
Control*inner tooth side	0				
Control*buccal tooth side	0				
UCT/LCT* TOOTH SIDE					0.1628
UCT*inner tooth side	-0.367	0.2628	-0.8826	0.1485	
UCT*buccal tooth side	0				
LCT*inner tooth side	0				
LCT*buccal tooth side	0				

Table 5: Coefficients, SE, and 95% CI of variables included in the model regarding sulcus gingivalis ≥ 1mm

			95% Confidence Limits		
	Coefficient	SE	Lower Bound	Upper Bound	*global* *P*-value
AGE	0.02039	0.02619	-0.03099	0.07177	0.4364
GROUP					0.0004
PPID	1.1961	0.3735	0.4632	1.929	
Control	0				
UPPER CT/ LOWER CT					0.0435
UCT	0.806	0.3499	0.1195	1.4925	
LCT	0				
TOOTH SIDE					0.0018
palatinal/lingual tooth side	-1.1447	0.4219	-1.9725	-0.3168	
buccal tooth side	0				
GROUP*UCT/LCT					0.0033
PPID*UCT	-1.1734	0.399	-1.9562	-0.3906	
PPID*LCT	0				
Control*UCT	0				
Control*LCT	0				
GROUP*TOOTH SIDE					0.0942
PPID*inner tooth side	0.669	0.3994	-0.1147	1.4526	
PPID*buccal tooth side	0				
Control*inner tooth side	0				
Control*buccal tooth side	0				
UCT/LCT* TOOTH SIDE					0.2993
UCT*inner tooth side	0.3795	0.3655	-0.3376	1.0966	
UCT*buccal tooth side	0				
LCT*inner tooth side	0				
LCT*buccal tooth side	0				

Table 6: Coefficients, SE, and 95% CI of variables included in the model regarding gingival texture changes at interdental gingival positions

			95% Confidence Limits		
	Coefficient	SE	Lower Bound	Upper Bound	*global* *P*-value
AGE	0.02107	0.01845	-0.01513	0.05728	0.2537
GROUP					0.0221
PPID	0.354	0.2537	-0.1438	0.8518	
Control	0				
UPPER CT/ LOWER CT					0.0590
UCT	-0.5389	0.2245	-0.9793	-0.09842	
LCT	0				
TOOTH SIDE					<.0001
palatinal/lingual tooth side	-0.8872	0.2316	-1.3417	-0.4326	
buccal tooth side	0				
GROUP*UCT/LCT					0.9892
PPID*UCT	-0.00349	0.2587	-0.5111	0.5041	
PPID*LCT	0				
Control*UCT	0				
Control*LCT	0				
GROUP* TOOTH SIDE					0.6365
PPID*inner tooth side	0.1224	0.259	-0.3857	0.6305	
PPID*buccal tooth side	0				
Control*inner tooth side	0				
Control*buccal tooth side	0				
UCT/LCT* TOOTH SIDE					0.0218
UCT*inner tooth side	0.5923	0.2577	0.08651	1.098	
UCT*buccal tooth side	0				
LCT*inner tooth side	0				
LCT*buccal tooth side	0				

Table 7: Coefficients, SE, and 95% CI of variables included in the model regarding gingival margin irregularity

			95% Confidence Limits		
	Coefficient	SE	Lower Bound	Upper Bound	*global* *P*-value
AGE	0.1277	0.04759	0.03384	0.2215	0.0079
GROUP					0.0404
PPID	0.4303	0.6349	-0.8213	1.682	
Control	0				
UPPER CT/ LOWER CT					0.4632
UCT	1.0556	0.549	-0.02674	2.138	
LCT	0				
TOOTH SIDE					<.0001
palatinal/lingual tooth side	-1.8284	0.6158	-3.0425	-0.6144	
buccal tooth side	0				
GROUP*UCT/LCT					0.5641
PPID*UCT	-0.399	0.6905	-1.7604	0.9624	
PPID*LCT	0				
Control*UCT	0				
Control*LCT	0				
GROUP* TOOTH SIDE					0.0491
PPID*inner tooth side	1.3758	0.695	0.005578	2.7461	
PPID*buccal tooth side	0				
Control*inner tooth side	0				
Control*buccal tooth side	0				
UCT/LCT* TOOTH SIDE					0.0766
UCT*inner tooth side	-1.2235	0.6876	-2.5792	0.1321	
UCT*buccal tooth side	0				
LCT*inner tooth side	0				
LCT*buccal tooth side	0				

Table 8: Coefficients, SE, and 95% CI of variables included in the model regarding diastemata

			95% Confidence Limits		
	Coefficient	SE	Lower Bound	Upper Bound	*global* *P*-value
AGE	0.2107	0.02565	0.1604	0.261	<.0001
GROUP					0.2411
PPID	-0.4462	0.259	-0.9544	0.06203	
Control	0				
UPPER CT/ LOWER CT					0.0006
UCT	-0.7902	0.2837	-1.3469	-0.2335	
LCT	0				
GROUP*UCT/LCT					0.2512
PPID*UCT	0.3941	0.3433	-0.2796	1.0678	
PPID*LCT	0				
Control*UCT	0				
Control*LCT	0				

Table 9: Coefficients, SE, and 95% CI of variables included in the model regarding irregularities in the MGJ

			95% Confidence Limits		
	Coefficient	SE	Lower Bound	Upper Bound	*global* *P*-value
AGE	0.1391	0.06005	0.02052	0.2578	0.0218
GROUP					0.6979
PPID	1.0476	0.6669	-0.2698	2.3651	
Control	0				
UPPER CT/ LOWER CT					<.0001
UCT	2.9674	0.6949	1.5947	4.3402	
LCT	0				
GROUP*UCT/LCT					0.0529
PPID*UCT	-1.6622	0.8521	-3.3456	0.02125	
PPID*LCT	0				
Control*UCT	0				
Control*LCT	0				

Table 10: Coefficients, SE, and 95% CI of variables included in the model regarding plaque and peripheral caries

			95% Confidence Limits		
	Coefficient	SE	Lower Bound	Upper Bound	*global* *P*-value
AGE	-0.04121	0.01824	-0.07699	-0.00542	0.0241
GROUP					<.0001
PPID	-0.9442	0.2658	-1.4657	-0.4227	
Control	0				
UPPER CT/ LOWER CT					0.0036
UCT	-0.1535	0.2157	-0.5767	0.2698	
LCT	0				
TOOTH SIDE					<.0001
palatinal/lingual tooth side	-0.8602	0.2292	-1.3099	-0.4105	
buccal tooth side	0				
GROUP*UCT/LCT					0.0384
PPID*UCT	0.5997	0.2893	0.03207	1.1674	
PPID*LCT	0				
Control*UCT	0				
Control*LCT	0				
GROUP* TOOTH SIDE					0.0173
PPID*inner tooth side	-0.7111	0.2984	-1.2964	-0.1257	
PPID*buccal tooth side	0				
Control*inner tooth side	0				
Control*buccal tooth side	0				
UCT/LCT* TOOTH SIDE					0.0471
UCT*inner tooth side	0.5676	0.2856	0.007197	1.128	
UCT*buccal tooth side	0				
LCT*inner tooth side	0				
LCT*buccal tooth side	0				

Table 11: Coefficients, SE, and 95% CI of variables included in the model regarding periodontal pockets

			95% Confidence Limits		
	Coefficient	SE	Lower Bound	Upper Bound	*global* *P*-value
AGE	0.1376	0.02772	0.08319	0.192	<.0001
GROUP					0.0226
PPID	-1.124	0.3511	-1.813	-0.4349	
Control	0				
UPPER CT/ LOWER CT					0.0479
UCT	-0.74	0.3389	-1.4051	-0.07499	
LCT	0				
TOOTH SIDE					0.4087
palatinal/lingual tooth side	-0.532	0.3307	-1.1808	0.1169	
buccal tooth side	0				
GROUP*UCT/LCT					0.1664
PPID*UCT	0.5397	0.3897	-0.225	1.3045	
PPID*LCT	0				
Control*UCT	0				
Control*LCT	0				
GROUP* TOOTH SIDE					0.1399
PPID*inner tooth side	0.572	0.3872	-0.1878	1.3318	
PPID*buccal tooth side	0				
Control*inner tooth side	0				
Control*buccal tooth side	0				
UCT/LCT* TOOTH SIDE					0.6557
UCT*inner tooth side	0.1712	0.3839	-0.5821	0.9245	
UCT*buccal tooth side	0				
LCT*inner tooth side	0				
LCT*buccal tooth side	0				
